# From Amorphous Silicones to Si-Containing Highly Ordered Polymers: Some Romanian Contributions in the Field †

**DOI:** 10.3390/polym13101605

**Published:** 2021-05-16

**Authors:** Maria Cazacu, Carmen Racles, Mirela-Fernanda Zaltariov, Mihaela Dascalu, Adrian Bele, Codrin Tugui, Alexandra Bargan, George Stiubianu

**Affiliations:** Department of Inorganic Polymers, “Petru Poni” Institute of Macromolecular Chemistry, Aleea Gr. Ghica, Voda 41A, 700487 Iasi, Romania; raclesc@icmpp.ro (C.R.); zaltariov.mirela@icmpp.ro (M.-F.Z.); amihaela@icmpp.ro (M.D.); bele.adrian@icmpp.ro (A.B.); tugui.codrin@icmpp.ro (C.T.); anistor@icmpp.ro (A.B.); george.stiubianu@icmpp.ro (G.S.)

**Keywords:** silicones, Romania, polymer morphology, siloxane-organic copolymers, highly ordered structures, coordination polymers

## Abstract

Polydimethylsiloxane (PDMS), in spite of its well-defined helical structure, is an amorphous fluid even at extremely high molecular weights. The cause of this behavior is the high flexibility of the siloxane backbone and the lack of intermolecular interactions attributed to the presence of methyl groups. These make PDMS incompatible with almost any organic or inorganic component leading to phase separation in siloxane-siloxane copolymers containing blocks with polar organic groups and in siloxane-organic copolymers, where dimethylsiloxane segments co-exist with organic ones. Self-assembly at the micro- or nanometric scale is common in certain mixed structures, including micelles, vesicles, et cetera, manifesting reversibly in response to an external stimulus. Polymers with a very high degree of ordering in the form of high-quality crystals were obtained when siloxane/silane segments co-exist with coordinated metal blocks in the polymer chain. While in the case of coordination of secondary building units (SBUs) with siloxane ligands 1D chains are formed; when coordination is achieved in the presence of a mixture of ligands, siloxane and organic, 2D structures are formed in most cases. The Romanian research group’s results regarding these aspects are reviewed: from the synthesis of classic, amorphous silicone products, to their adaptation for use in emerging fields and to new self-assembled or highly ordered structures with properties that create perspectives for the use of silicones in hitherto unexpected areas.

## 1. Context and Early Romanian Achievements in the Field of Silicones

Research in the field of silicones in Romania started more than 50 years ago and has addressed either fundamental or applied aspects, depending on the state policies of specific governments (Figure S1 in supplementary material). Following the development of its own technology for obtaining chlorosilanes, basic monomers for synthesis of silicones, research has focused on silicone cycles, oils, fats, emulsions, rubbers and resins, as well as polysilanes. More than 45 applied research works and technological processes were elaborated at laboratory and pilot scale, which were put into operation in 1985 at Petrochemical Plant Borzesti, thus laying the foundations for a national silicone industry, with four pilot stations and two silicone production lines. As a result of the applied research in collaboration with industry, trademarks for 10 silicone products under the name MOLDOSIL and 54 patents (e.g., [1,2,3,4,5,6,7,8,9,10]) were granted between 1970 and 1992. The main fundamental research approached since the 90s has been aimed at: (a) techniques for polymerization and copolymerization of cyclosiloxanes (homogeneous catalysis, with thermolabile catalysts, heterogeneous catalysis, kinetics, molecular weight control, etc.) [11,12,13,14,15,16,17]; (b) introduction of organic functions on silicon by various mechanisms (hydrosilylation, condensation, dehydrocoupling, thiol-ene addition, etc.), study of their effects on the properties of the resulting compounds and evaluation of their functionality as blocks for siloxane-organic copolymerization [15,18,19,20,21]; (c) obtaining of block [22,23] or segmented [24,25,26,27] siloxane-organic copolymers with different internal functions (silyl ether, silyl ester, urethane, imide, amide, imine, etc.) showing different properties (self-assembling ability, liquid crystals, redox or surface activity, pH sensitivity, controlled hydrolytic degradability, etc.) emphasized by specific investigations; (d) silicone crosslinking systems and mechanisms (peroxide, hydrosilylation, condensation, thiol-ene addition) for obtaining rubbers and other materials with applications in medicine, dentistry, construction, optoelectronics, etc.) [28,29,30]; (e) advanced polycarbosilane and polysilane structures [31,32]; and (f) series of hybrid polymeric materials, such as crosslinked siloxane-urethane, siloxane-chitosan or cellulose-siloxane structures [24,33,34,35,36,37], composites (silicone/silica, silicone/silica/titania, silicone/silica/Fe_2_O_3_) adapted-by varying molecular weight, component ratios or crosslinking procedures to meet the requirements for certain applications (dentistry, actuators, etc.) [28,29]. For the first time, lignin was incorporated into silicone matrices to obtain adhesive composites applicable in masonry and water-repellent materials [38,39].

From 2010 to the present, research has been carried out in the field of silicones, both fundamental and oriented towards target applications, approaching mainly two directions: (a) The transition from passive silicones to active ones that can be recycled, repaired and can reversibly and repeatedly change one or more characteristics (shape, size, color) under the action of an external stimulus, such as temperature change, pressure, applying an electric, magnetic or optical field, and changing the humidity or polarity of the environment. Such materials are of interest for the construction of adaptive systems for a wide range of applications in the automotive, medical technology, white goods, mechanical engineering, consumer sectors, et cetera; and (2) design of organic-inorganic hybrid ligands and obtaining complex structures with metals having different dimensions (0D, 1D, 2D, 3D), which constitute a remarkable class of compounds with special properties allowed by the presence in the structure of highly flexible and hydrophobic silicone motifs. The results are found in publications whose distribution on different categories of compounds and on different centers in Romania are represented in Figures S1 and S2 (in supplementary material), respectively.

**Figure 1:**
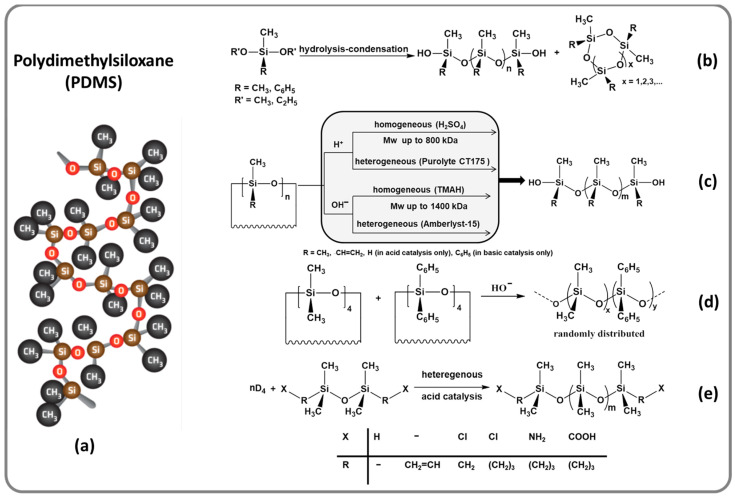
A free polydimethylsiloxane chain fragment as a representative structural motif for silicones (**a**) and main pathways approached for obtaining oligo- or polydiorganosiloxanes: hydrolysis-condensation of dialkoxysilanes (**b**); ring-opening polymerization of cyclosiloxanes (**c**); ring-opening copolymerization of diorganocyclosiloxane mixtures (**d**); diorganocyclosiloxane equilibration with 1,3-bis(functionalized)disiloxanes (**e**).

The main interest was to obtain polydimethylsiloxane, PDMS, (Figure 1a), which is the basis of most silicone materials. Multiple studies have been conducted by the authors on the hydrolysis-condensation of dichloro- or dialcoxy-functionalized diorganosilanes, to prepare and isolate either linear or cyclic oligodiorganosiloxanes having identical or different organic groups attached to the silicon atom, in order to be used as is or in further chemical transformations (Figure 1b). To obtain polydiorganosiloxanes in a wide range of molecular weights, ionic ring-opening polymerization en masse was performed, by either homogeneous or heterogeneous catalysis [11,12,13,14,15,16,40] (Figure 1c). The reaction conditions were optimized and the performances and limits of each system were assessed; useful information for choosing the appropriate pathway in each concrete case. Thus, both syntheses in acidic (H_2_SO_4_) and in basic (tetramethylammonium hydroxide (TMAH) or silanolate) medium were approached, depending on the nature of the substituents from the silicon atoms, by either homogeneous or heterogeneous catalysis. The use of TMAH as a transient catalyst is an efficient way to obtain very high molecular weight polydiorganosiloxanes having low polydispersity index, in a single step. Thus, ultra-high molecular weight polydimethylsiloxane (UHMW-PDMS) with *M*_w_ of order 1400 kDa and polydispersity index, PDI = 1.2 was obtained [41]. This procedure was also implemented for the synthesis of the dimethyldiphenylsiloxane copolymers (Figure 1d) and other polysiloxanes functionalized with basic groups [16,42,43]. Significant research efforts have focused on using synthetic ion exchangers as catalysts in the equilibrium reactions involving siloxane bonds, including kinetic and optimization studies. The procedure was adapted for the synthesis of diorganosiloxane oligomers, polymers, and copolymers, as well as oligo- and polysiloxanes end- or side-modified with polar or reactive groups (Figure 1e) [5].

## 2. Classic Amorphous Silicones Renewed for Emerging Applications

Polydimethylsiloxane (PDMS) is the basis of the vast majority of silicone products that have found use in almost all areas of human activity, from cosmetics to the nuclear or aerospace industry [44]. Although last year (2020) the scientific community marked 80 years since the first direct synthesis of silicones, they still enjoy great interest, both scientific and applicative. The versatility of silicone chemistry allows their chemical modification to give them new capabilities, while maintaining their unique, useful properties. PDMS has a backbone consisting of alternating silicon and oxygen atoms, with non-polar organic groups (methyl) [45] attached to the silicon atoms. The freedom of rotation of the methyl groups conferred by the flexibility of the siloxane backbone and the long length of the Si-CH_3_ bond (1.88 Å) make these groups form a “shield” around the chain [46,47] (Figure 1a). This makes intermolecular interactions almost non-existent, the material itself being hydrophobic and permeable to gases, with low viscosity, solubility parameters and low glass transition temperature. A property also resulting from this structure is very low surface tension. The liquid PDMS surface tension is 21 mN/m at 25 °C [48], while its critical surface tension [49] is 24 mN/m (compared to those of mineral oil and deionized water which are 30.4 and 72.0 mN/m, respectively) at the same temperature [50]. This makes the silicones spread very easily, distinguished by their ability to form temporary films and thin coatings to more substantial durable films or with self-leveling and adhesive capacities as stand-alone sheets of different sizes and thickness, from a few micrometers to a few millimeters [46,47]. One application that is based on this property is the formation of free standing, flexible submicrometric films of interest as active elements in certain devices, such as dielectric elastomer transducers (DETs). Dielectric elastomers (DEs), three-dimensional networks of long and flexible polymer chains, are soft active materials showing promising properties that mimic natural muscle for use in advanced robotics and smart prosthetics, as well as in haptic and microfluidic devices [51]. They enjoy great interest due to their inherent flexibility, large strain, high efficiency, high energy density, and fast response of the material [52].

**Figure 2:**
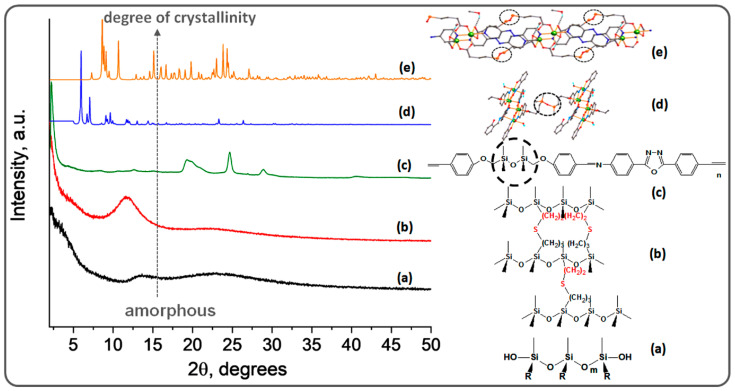
Modification of the X-ray diffraction pattern with the increase of the weight of the organic segment in hybrid structures: (**a**) a linear, high molecular weight PDMS-α,ω-diol [41]; (**b**) a crosslinked PDMS having thioether bridges between chains [53]; (**c**) a segmented poly(siloxane-azomethine) [54]; (**d**) 1D coordination polymer consisting of hexanuclear manganese(III) salicylaldoximate complex units connected by 1,3-propyltetramethyldisiloxane bridges [55]; 2D di-manganese coordination polymer with siloxane spaced dicarboxylic acid and 4,4’-azopyridine as co-ligands [56]. (**a**,**c**)—CuKα-emission (λ = 1.54059 Å, Rigaku Miniflex 600 diffractometer), (**d**,**e**)—graphite-monochromated Mo-Kα radiation (λ = 0.71073 Å, Oxford-Diffraction XCALIBUR E CCD diffractometer); the dotted circles mark the siloxane fragments within the hybrid structures.

Another property arising from the lack of intermolecular interactions and the high flexibility of the chains is the disorganization that gives a strong amorphous character to silicones and some of the derived materials (Figure 2a,b).

### Electromechanically Active Silicone Elastomers

**Figure 3:**
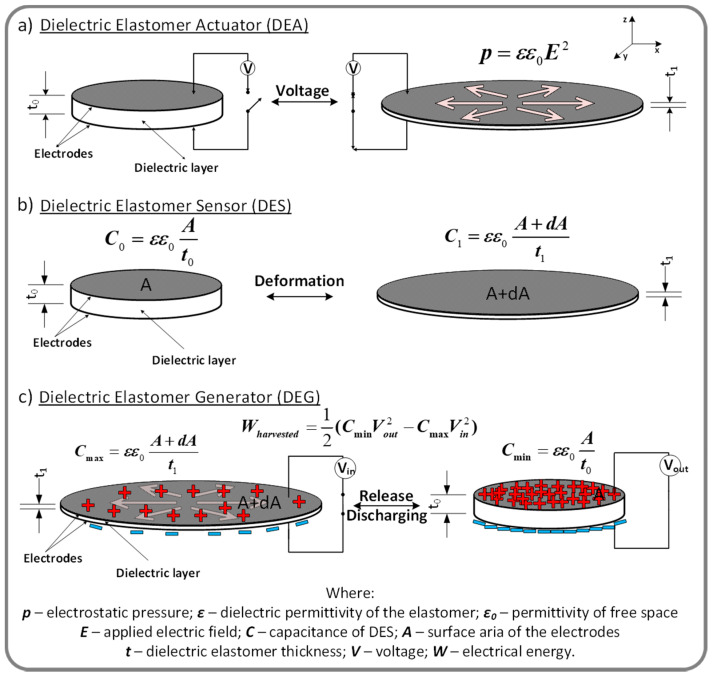
The principle of operation of dielectric elastomer transducers (DETs) as: (**a**) actuators (DEA); (**b**) sensor (DES); (**c**) generator (DEG).

Dielectric elastomers (DEs) are a class of electroactive polymers (EAPs) widely used as active elements in soft transducers (actuators, sensors and generators) [57]. Such a transducer consists in principle of a highly deformable dielectric elastomer film sandwiched between compliant electrodes. When a voltage is applied to the electrodes, the electrostatic force squeezes the dielectric elastomer film expanding it laterally due to the elastomer incompressibility (dielectric elastomer actuator, Figure 3a) [58]. Two mechanisms underlie the electrical action, Maxwell stress (caused by coulombian interaction between oppositely charged electrodes) and electrostriction (based on small displacements of dipoles from their equilibrium positions under an applied electric field [59]. However, although it occurs in all dielectrics, in the case of dielectric elastomers, the electrostriction contribution is low, often being ignored, especially in the case of non-polar polymers such as silicones [60].

Dielectric elastomers can also be used for measuring mechanical deformations, such as pressure, strain, shear and torsion acting as soft capacitive sensors (dielectric elastomer sensor, Figure 3b) [61,62]. Another important functionality of DEs, is their ability to work as generators by converting mechanical to electrical energy (Figure 3c) [63].

Since the discovery of the basic principles in 1990 and the first application of electroactiv**e** elastomers in the field of robotics, followed by the first use of DEGs for wave energy conversion (WEC) in 2001 and the first fully elastomeric WEC in 2012 [64] to the present, the field is constantly expanding and developing. The electromechanical transducers based on dielectric elastomers (DETs) show many benefits in comparison with traditional piezoelectric, magnetic, pneumatic or hydraulic transducers that are in general bulky, rigid, expensive and often unsuitable due to their incompatibility with soft or complex-shaped objects. The thin, homogeneous, soft and flexible polymer film is the key to the low voltage operation of multilayer DETs to qualify as artificial muscles.

Although such DETs are of interest for a growing number of applications (adjustable optics, microfluidics, soft robotics and haptic devices) [65,66,67,68,69] and technically feasible, there are still some challenges to be addressed: rapid and reproducible production of elastomeric membranes, the ability to self-repair defects of a certain level, realization and deposition of extensible electrodes, ensuring their good compliance with the dielectric film, et cetera [70]. Thus, although the dielectric is the active element of a DEG, the electrodes are also at the core of the device’s performance: they must be conductive, soft and sustain large deformations while remaining conductive, and they must also be able to do so for millions of cycles. The main requirements for dielectric elastomers consist mainly of large actuation strain, low modulus and low elastic losses, high dielectric strength, fast response, low weight, low costs, recyclability, et cetera [71]. Of the types of elastomers tested for DETs purpose over time, only silicones and acrylics have proven to be competitive. Silicones meet most of these requirements, being primarily highly compliant and capable of large deformations due to their high flexibility [72], having shear modulus values between 100 kPa and 3 MPa, and low loss tangent, tan σ << 0.001 [73]. In addition, they can operate in a wide temperature range, are easy to handle and process in the form of films of different thicknesses [74,75]. Silicones also have advantages in terms of resistance to the environment (humidity, ozone, oxygen, sunlight, marine environment, etc.) and weathering (Table 1), but they have the drawback that they are not biodegradable, while recycling processes are complicated [76].

**Figure 4:**
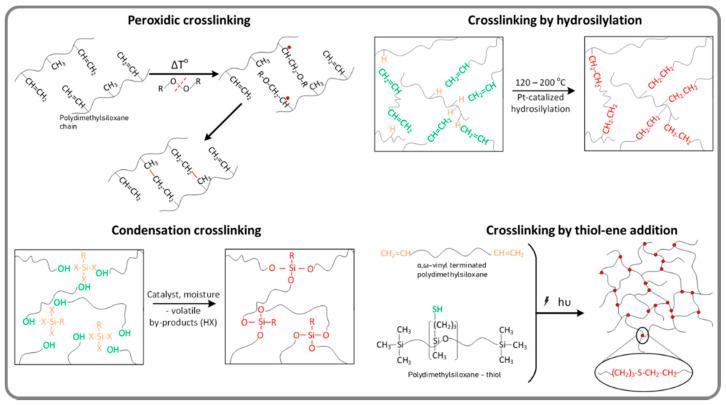
Approaches for increasing the performance of dielectric elastomers using different cross-linking pathways: peroxide, condensation, hydrosilylation, dehydrocoupling, thiol-ene addition [53].

Based on the above and due to their appropriate mechanical properties (low modulus and high elongation), silicones are among the most used polymers in DETs. Their properties can be easily tuned by the preparation strategy: proper choice of the molecular mass and microstructure of the polymer matrix; adding of more or less active fillers, whether these are incorporated in the polymeric matrix (*ex situ*) or generated *in situ*; crosslinking mode (through the side or ending functional groups) or mechanism (condensation, radicalic or by hydrosilylation). Thus, it was possible to improve the performance of silicone elastomers by manipulating parameters, such as the molecular weight of the polymer and the crosslinking pattern, without other additions or chemical changes. Polydimethylsiloxanes with molecular weights from several tens of thousands to over one million Da have been obtained [41]. They were crosslinked by different chemical pathways: peroxide, condensation, hydrosilylation, dehydrocoupling and thiol-ene addition (Figure 4) [53] obtaining soft to ultra-soft silicones, with Young’s moduli generally less than 0.1 MPa and elongation at break of over 1000%, and good values for elastic recovery and stress decay [41].

**Figure 5:**
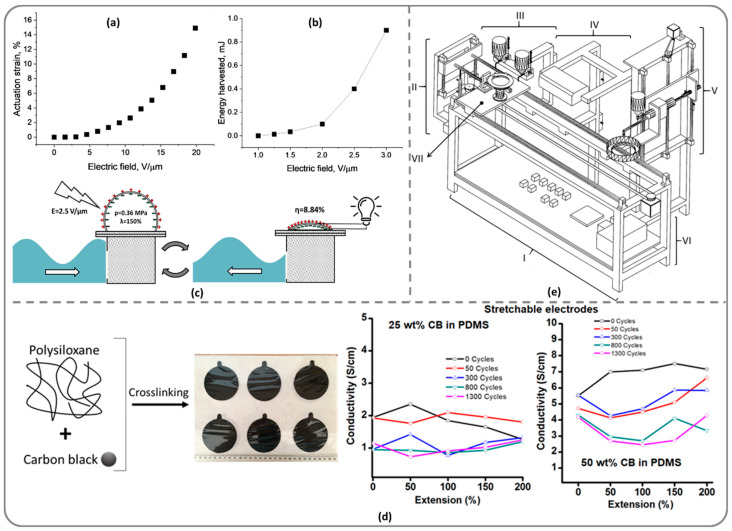
Own performances obtained with dielectric silicone elastomer transducers: (**a**) actuation showed by a DEA with single-layer silicone film based on PDMS (Mw = 80,000 Da) containing 8.4% vinyl groups crosslinked by thiol-ene addition with a PDMS with Mw = 35,000 Da containing 16.5% SH group along the chain [53]; (**b**) energy harvesting with a DEG based on three layers of silicone elastomer and stretchable electrodes also based on silicone embedding 25 wt% carbon black [79]; (**c**) energy collected with a DEG based on silicone filled with 2 wt% TiO_2_ nanotubes and carbon black electrode [80]; (**d**) conductivity performances of stretchable electrodes based on PDMS incorporating 25 wt% carbon black within the PDMS matrix [81]; (**e**) automatic installation for stacked DET manufacturing (block I—stainless steel rigid skeleton, block II—automatic drop of the circular mask, block III—two spray guns that allow the deposition of the electrode solution and the silane, block IV—UV lamp, block V—polymer dispenser, block VI—electronic parts, block VII—left-right mobile rotating disc on stainless steel plate) [82].

By properly designing polysiloxanes in terms of structure, molecular weight and crosslinking pattern, it was found that a silicone elastomer based on PDMS (*M*_w_ = 80,000 Da) containing 8.4 mol.% vinyl groups crosslinked by thiol-ene addition with a PDMS (*M*_w_ = 35,000 Da) containing 16.5 mol.% SH group along the chain, without other additions, incorporated in a DEA developed a lateral actuation of 15% when applying an electric field E = 20 MV/m (Figure 5a) [53]. A DEG built from three circular layers of common silicone with a diameter of 120 mm and coaxial circular stretchable electrodes of 60 mm diameter also based on silicone but embedding 25 wt.% carbon black was able, at 200% strain, to harvest almost 1 mJ (Figure 5b) [79]. An energy harvesting efficiency of η = 8.84% was achieved with a DEG based on silicone filled with 2 wt.% TiO_2_ nanotubes and carbon black electrodes (Figure 5c) [80]. By incorporating carbon black [81] or carbon nanotubes within the PDMS matrix, stretchable electrodes were obtained, several conductivity performances of these being showed in Figure 5d.

However, silicones suffer from low value of dielectric permittivity, requiring relatively high electric fields (E > 100 MV/m) on the electrodes for actuation [83,84,85,86]. Therefore, significant efforts are being made to improve this parameter. One of the ways to improve the performance of silicone elastomers in terms of dielectric permittivity against the background of their other properties of interest is to attach polar groups [87]. This can be done on monomers (silanes or cyclosiloxanes) or by post-functionalization of polymers containing certain reactive groups, generally Si-H, Si-CH = CH_2_ or RX (R: alkyl, X: NH_2_, halogen, SH) by hydrosilylation, thiol-ene addition or substitution reactions. While some of these groups are themselves of interest due to polarity, their modification can lead to more complex structures. Thus, groups such as cyan, epoxy, benzaldehyde, dispersed Red1 (DR1) [88], acetate, carboxyl and chloride [89] were attached to increase the dielectric permittivity of silicones and indeed this effect has been achieved, and more. Although they have very good dielectric properties, due to phase separation, polar silicones most often show weak mechanical properties, which limit their applicability in electromechanical devices. An alternative solution has been considered, polysiloxanes carrying CN-propyl or Cl-propyl groups were processed as submicron particles stabilized by either full organic [90] or a specially designed siloxane surfactant [91], cross-linked within the formed particles and used as fillers in a high molecular weight polydimethylsiloxane matrix [90,91]. Soft elastomers with Young’s moduli of 0.12–0.5 MPa, depending on the filler’s structure, dielectric strength up to 63 MV/m and dielectric permittivity up to 4.7 at 10^4^ Hz were obtained. High apparent piezoelectric coefficient (*d*_33_) was measured by piezoresponse force microscopy (PFM) without poling, at ambient temperature, while variation of *d*_33_ with stretching was observed, in correlation with morphological aspects [90]. Elastomeric composites with improved mechanical properties (higher elongation and tensile strength) and electromechanical response (higher *ac*tuation) were obtained. Polyazomethine (PAZ) submicron particles (either containing siloxane moieties, or not) have also been used as fillers for silicone-based composites [92]. Polar particles were obtained by polycondensation reactions performed in organic solvents in the presence of an amphiphilic siloxane oligomer. Elastomers with increased dielectric permittivity relative to pure polydimethylsiloxane elastomer, excellent stretchability with elongations in excess of 500% and up to 800% and low Young’s moduli were obtained by this approach.

Much research has also been done on the preparation of elastomeric silicone composites by incorporation in a silicone matrix with different parameters (molecular weight, functionality, degree of crosslinking, etc.) of other active fillers capable of increasing its dielectric permittivity. Fillers of different types were tested with this aim. For example, ceramic fillers (BaTiO_3_ [93,94,95], TiO_2_ [80]) having different morphologies and inorganic ones (*in situ* generated silica [96]) were used. Studies have shown that the success of such an approach depends primarily on the degree of dispersion and compatibility of the filler with the matrix. For this, an adequate surface treatment is required, which also ensures a reasonable dielectric strength for the resulting material. In the case of the use of BaTiO_3_ having different morphologies, the particles with high aspect ratio were found to be more adequate, an addition of only 5 wt.% BaTiO_3_ leading to an increase of about three times of the relative dielectric permittivity compared to the value measured for the silicone matrix [94]. The electromechanical sensitivity and actuation strain, calculated from mechanical and dielectric data, proved to be the highest in the case of acicular particles, also being high compared to those reported in the literature (electromechanical sensitivity, β ~ 400 MPa^−1^ and lateral actuation strain Sx ~ 23%), thus promise for a good effective electromechanical response exists [94]. TiO_2_ nanotubes were first properly treated on the surface with hexamethyldisilazane and incorporation (5 wt.%) in the silicone matrix led to a dielectric elastomer with dielectric permittivity of 7.7 at 0.1 Hz and dielectric strength of 60 MV/m. A synergy between the increased dielectric permittivity and a low Young’s modulus reflected a good electromechanical response of the composites, resulting in 8.84% efficiency for 400 V input voltage and 150% balloon-like elongation (Figure 5c) [80].

Besides the ceramic particles, less studied materials as permittivity enhancers for PDMS are metal (Mn, Fe, Cr) complexes of salen-type Schiff bases with tetramethyldisiloxane spacers [97]. The resulted composites showed a slight decrease of elastic properties but an increase in dielectric permittivity of up to 100%, while the electromechanical sensitivity was almost double compared with the reference sample. Cu-, Co-, and Ni-atranes [98] or a 1D coordination polymer [99], were also tested as fillers for silicones. While in the latter case, a dielectric permittivity value of 4.6 was obtained at a load of 3 wt.% filler, regardless of the frequency, in the first case, remarkably high values were registered for dielectric permittivity but, unfortunately, also for dielectric loss, which makes these composites fall more into the semiconductor field. Iron oxide nanoparticles are another category of inorganic fillers studied as permittivity enhancers for polysiloxanes [100,101]. Spherical ferrihydrite and nanorods of goethite were prepared and subsequently incorporated into the PDMS matrix [101]. The nanocomposites prepared with spherical nanoparticles showed permittivity values up to 5 at 1 kHz and electromechanical sensitivity comparable to that of composites with ceramic fillers.

The dielectric permittivity of polysiloxanes can also be increased by incorporating conductive fillers at concentrations below the percolation threshold. There are two strategies of using these fillers as permittivity enhancers, either the concentration of the filler is kept below the percolation threshold or the fillers are coated with surfactants or other materials to avoid this phenomenon. Composites based on silicones and conductive fillers are also being studied as compliant electrodes. Considering that the carbon black particles represent the cheapest powder that can be used as a conductive filler, a rubber electrode was fabricated by incorporating 25 wt.% of carbon black into a high molecular weight PDMS [79,81]. The electrical conductivity was about 0.1 S·cm^−1^ at 150% strain, remaining almost unchanged even after a few hundred stretching cycles (Figure 5d). By increasing the carbon black load to 50%, the conductivity increases considerably but the cyclic operation performance decreases. An automatic installation for the alternating deposition of rubbery dielectric and electrode layers for obtaining stacked DETs has been patented [82] (Figure 5e). This consists of several blocks (I–VII), which allows obtaining the alternating deposition of dielectric and electrode layers as a stacked DET [101]. A stainless-steel rigid block (I) sustains all other blocks, while a mobile block (VII), that mainly consists of a rotating disc, moves horizontally in four steps to obtain two consecutive sacrificial layers, two electrodes and one active layer, as follows. In the first step, a silane solution is sprayed with a gun on the rotating disc from block VII, under block III. In the second step, a certain amount of polymer is poured on the disc after block VII moves under block V and spin-coated to obtain the desired thickness. The UV crosslinking of the thin polymer layer occurs under block IV in the third step. The first electrode is obtained by moving block VII beside block II and under block III. At this step, the fourth, a circular mask drops on the rotating disc, the disc rotates with the mask and simultaneously the electrode solution is sprayed with a gun onto the disc. The mask allows obtaining the circular shape of the electrode. These steps are repeated to obtain the active layer, the second electrode and the final sacrificial layer. Several performances obtained with such formulations and arrays are showed in Figure 5e.

## 3. Siloxane-Siloxane and Siloxane-Organic Copolymers

The lack of intermolecular interactions between chains causes PDMS to be amorphous in normal conditions (Figure 2a) and to even flow at very high molecular weights (even those of the highest molecular weight obtained, more than a million Da [102,103]). It would be expected that the partial replacement of methyl groups with other organic groups capable of developing intermolecular interactions would produce significant changes in the behavior of the polymer. In reality, the situation is much more complicated. The difference in polarity between the methyl group and any other more polar organic group leads to phase segregation [20]. This could be avoided or diminished by the synthesis of perfectly alternating copolymers. However, they are almost impossible to obtain due to the equilibrium nature of the reaction for obtaining polysiloxanes [14]. An alternative would be the reactions under kinetic control, but in this case they are also limitedly imposed by the adequacy of the substituents to such reactions which are conducted mainly in a basic environment.

**Figure 6:**
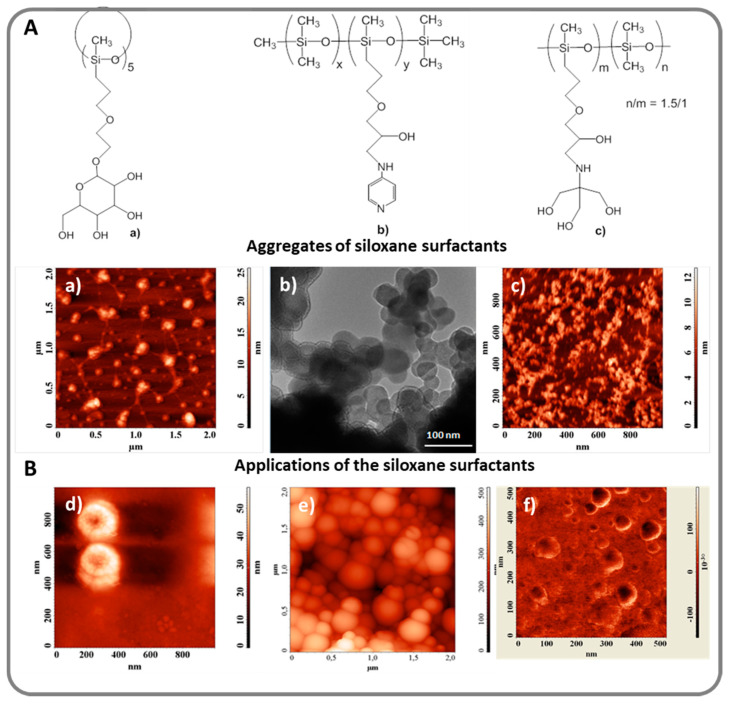
(**A**): Examples of siloxane surfactants and their aggregation patterns. (**a**,**c**) Atomic Force Microscopy (AFM) images, (**b**) Transmission Electron Microscopy (TEM) image) [106,108,109]. (**B**) Selected applications of original siloxane surfactants (exemplified in AFM images); (**d**) micellar solubilization of drugs [110]; (**e**) nanoreactors for polymer synthesis [111]; (**f**) stabilization of metal oxide nanoparticles in water [112].

However, phase separation can be exploited, a major application of this being the self-assembly of amphiphilic materials, which is the motor for their behavior as surface active compounds, stabilizers or nanoreactors. Siloxane-based surfactants are very interesting in this context. Based on the pronounced hydrophobicity of the dimethylsiloxanes, the surface tension of water and certain organic solvents is seriously diminished, reaching values of ca. 20 mN/m, at low concentrations. In terms of morphology, the preferred type of aggregates are vesicles [104,105,106,107], which opens interesting possibilities for drug delivery systems and cosmetic formulations. Aggregates of siloxane surfactants containing glucose [108], amino-pyridine [109] or tromethamol [106] hydrophilic groups are exemplified in Figure 6A, while in Figure 6B some of the applications proposed for siloxane surfactants are illustrated. Encapsulation of a non-soluble drug in the surfactant’s vesicles (Figure 6d) [110], stabilization of polyazomethine nanoparticles synthesized directly in the aqueous micelles (Figure 6e) [111] or transfer of super-paramagnetic iron oxide nanoparticles, SPIONs, into water (Figure 6f) [112] have been demonstrated.

**Figure 7:**
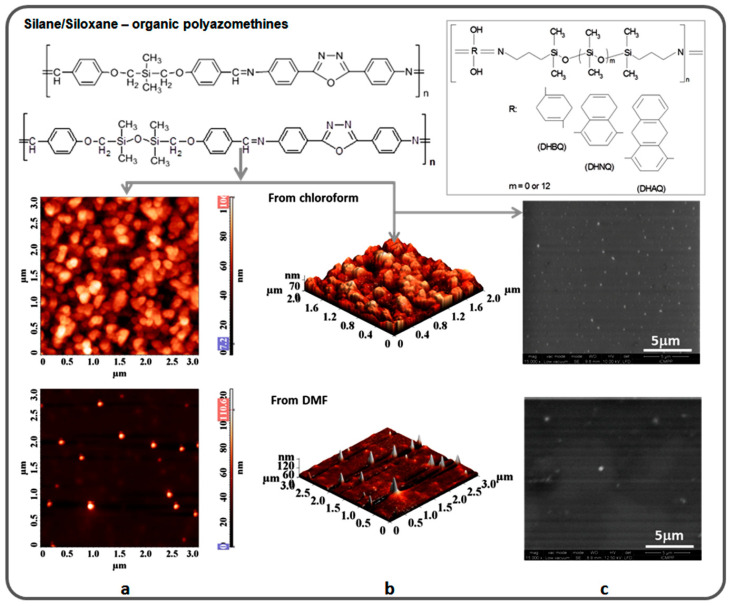
Examples of segmented silane/siloxane-organic copolymers [54,114,115,116] and illustration through 2D and 3D AFM (**a**,**b**) and TEM (**c**) images of their self-assembly that changes depending on the polarity of the environment.

Block or segmented siloxane-organic copolymers also show phase separation with the formation of micelle or vesicle-like aggregates, this being a common phenomenon in block-copolymers formed from incompatible partners [113]. This phenomenon occurs even for small siloxane segments combined with practically any organic fragment, due to the afore-mentioned peculiarities of polysiloxane (pronounced hydrophobicity, very low surface energy, high chain flexibility, very low negative glass transition temperature). A wide range of siloxane-organic copolymers with organic blocks having a variety of internal functions (sulfone, amide, imide, urea, urethane, oxadiazole, silyl ether, silyl-ester, etc.) was obtained, many of which showed evidence of self-assembly capacity in selective solvents [24]. Thus, although in some cases, phase separation may jeopardize material properties, it is a tool for self-assembly in bulk or in selective solvents, a behavior of interest for nanotechnology. An example is shown in Figure 7, where crystallization from one solvent and formation of micelles in another were observed for amphiphile polysiloxane copolymers. The increased degree of ordering induced by the presence of polar organic blocks or bridges was also proved by the appearance of peaks in the X-ray diffractogram of the compound (Figure 2c).

**Figure 8:**
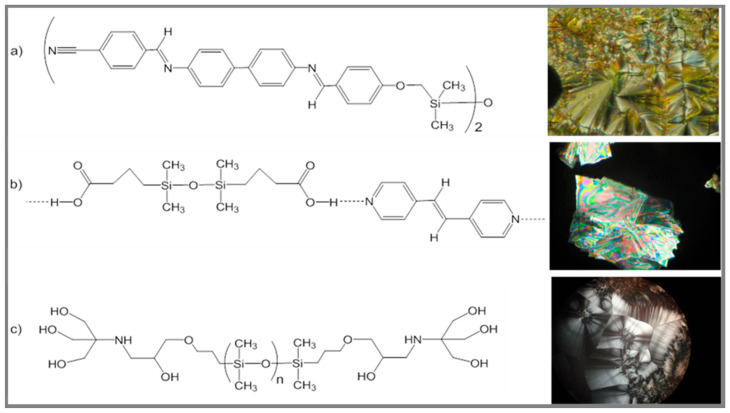
Polarized light optical microscopy images of siloxane-containing liquid crystals: **a**) a siloxane azomethine with smectic A focal conic texture at 330 °C (magnification 400x); **b**) an H-bond supramolecular polymer and its smectic crystal texture at 40 °C (magnification 200x); **c**) an amphiphilic siloxane oligomer forming LC phase at room temperature [124].

In terms of molecular organization, liquid crystals (LCs) are intermediary state materials, between disordered isotropic liquids and highly ordered 3D solid crystals [117], exhibiting long-range order. Siloxane moieties (short segments, long chains, cycles) are often used in LCs as flexible spacers or as the main backbone, in combination with unnumbered mesogenic groups. The main reasons for using siloxanes instead of purely organic (hydrocarbon) counterparts derive from their bulkiness, high flexibility, chemical versatility and tendency of microphase separation [118,119,120]. Several general characteristics of the resulted LC materials are due to these specific behaviors of (poly)siloxanes: lower transition temperatures, larger mesophase range, tendency for formation of smectic mesophases and suppression of the nematic mesophase. In our group a large variety of siloxane-containing LCs has been reported, mainly of azomethine type, but also polyethers, polyesters and H-bond supramolecular polymers [119,121,122,123]. By varying the siloxane segment lengths and the structure of the adjacent organic flexible groups, tuning of the mesophase range and type, as well as large solubility domains can be achieved. Besides main chain and side chain linear liquid crystalline polymers, polysiloxanes are probably best known as components of elastomeric liquid crystals (LCEs). These ordered crosslinked materials combine the order and mobility of the LC phase with rubber-like elasticity. Some of their characteristics and applications have recently been reviewed [104,105,106,107,113]. Polarized light optical microscopy images of various types of siloxane-containing LCs are exemplified in Figure 8, for a low molecular compound, an H-bond supramolecular polymer and an oligomeric surfactant [124].

## 4. Highly Ordered Coordination Polymers Containing Silicon or Silicone Motif

**Figure 9:**
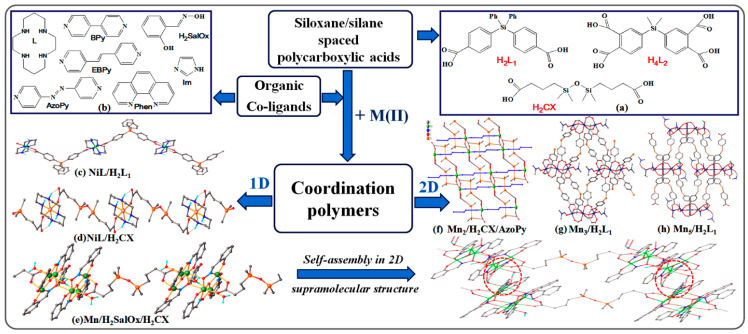
Carboxylic ligands containing silicon in the substrate (**a**) and organic co-ligands (**b**) used; 1D coordination polymer based on: diphenylsilane-spaced dicarboxylate (H_2_L_1_ ligand) coordinated to the NiL [131] (**c**), tetramethyldisiloxane-spaced dicarboxylate (H_2_CX ligand) coordinated to the NiL preformed complex [130] (**d**) and to oxime-bridged Mn^6+^ cluster [55] (**e**); Covalent 2D coordination polymers looking like nano-sheets consisting of: dimanganese coordinated by H_2_CX and 4,4’-azopyridine [56] (**f**); trinuclear (**g**) and pentanuclear (**h**) manganese coordinated with H_2_L_1_ [127].

Since 2010, the idea of attaching complexing groups to the silane or flexible siloxane substrates appeared. Thus, a series of Schiff bases developed mainly by the reaction of either 1,3-bis(3-aminopropyl)tetramethyldisiloxane or in-lab synthesized diamine 1,3-bis(amino-phenylene-ester-methylene)tetramethyldisiloxane by complexation with metals forming highly crystalline compounds. More than 30 such unique structures have been recorded in the crystallographic database, CCDC [125]. They are generally molecular compounds, some of which develop weak intermolecular interactions. However, highly ordered, crystalline polymeric covalent structures, where siloxane motifs alternate with coordinated metal blocks, were obtained with carboxylic ligands, 1,3-bis(carboxypropyl)tetramethyldisiloxane (H_2_CX) prepared for the first time in 1999 [15], bis(p-carboxyphenyl)diphenylsilane (H_2_L_1_), and bis(3,4-dicarboxyphenyl)dimethylsilane (H_4_L_2_) were prepared later [126] (Figure 9a). They were used alone to directly coordinate the metal ions [127,128,129] or complexes of the latter with vacancies [55,99,130,131] or in combination with ancillary organic ligands (imidazole [132,133], 4,4′-bipyridyl [134], 4,4′-azopyridine [56,132,135], 1,2-di(4-pyridyl)ethylene [134], 1,10-phenanthroline [126], etc.) (Figure 9b) and metal ions or clusters, which allow highly directional intermolecular interactions (metal-ligand, hydrogen bonds and π-π stacking interactions). As a result, dramatic changes occur in the X-ray diffraction pattern (Figure 2d,e). Twenty-three such structures were registered in the CCDC database.

The dimensionality (1D, 2D or 3D) and the structural motifs (linear or double chains, zig-zag, helix, square-grid, diamondoid, etc.) of the resulting coordination polymers depend on the molecular building blocks and on the synthesis conditions [136]. Different synthetic strategies are employed for obtaining coordination polymers, consisting of both conventional methods (slow solvents evaporation-saturation methods, slow diffusion of solvent or of reactants) and non-conventional methods, such as hydro/solvothermal, ultrasonic and microwave-assisted syntheses. Often, the same starting precursors can lead to different products, depending on the solvent, the molar ratio between the reactants, the metal center, the counter ion, temperature, pH, time, the cooling speed in hydro/solvothermal synthesis, et cetera. The assembly of the building blocks into a predictable architecture thus becomes a difficult task.

In our attempt to investigate the design and control of the self-assembly in coordination polymers with flexible or semi rigid (V-shaped) polycarboxylate ligands, especially those containing silane and siloxane units (H_2_L_1_, H_4_L_2_ and H_2_CX in Figure 9a), with the involvement of different organic co-ligands (Figure 9b), various metal complexes with interesting structures (Figure 9c–h) have been successfully isolated by template-directed synthesis, slow diffusion of solvents and reactants and solvothermal methods.

While the coordination bonds directly control the assembly of the building blocks, the weaker interactions (H bonds, π-π stacking and van der Waals interactions) consolidate the packing of the solid, crystalline structure. Such intermolecular interactions develop exclusively between the polar organic or inorganic blocks in the structure (Figure 9e), while the role of dimethylsiloxane or dimethyl-/diphenylsilane motifs remains one related to flexibility and stability, which affects transitions and thermal stability, solubility and hydrophobicity. The flexibility of this segment allows the adoption of both *cis*- [133,135], and *trans*-oide [55,133,135] conformations, in dependence on the coordination geometry of the secondary building units (SBUs) and H-bonding configuration. It was observed that while the complexation of metals with silicone ligands almost always leads to 1D structures [55,99,128,131,132,134], in the presence of an organic co-ligand 2D structures are often formed [56,127,132,133,134,135]. The 2D structures built with the H_2_Cx ligand are of interest from the perspective of 2D materials, presenting as hydrophobic nano-sheets with very weak interactions between them [56]. The carboxylate groups of the silicon-based ligands in these complexes also evidenced a versatile coordination behavior, in other words, ionic, monodentate, bidentate bridging, bidentate chelating or some combination of them.

Besides the highly ordered structure, in the obtained coordination polymers, the presence of the metal and of its coordination block induces specific properties such as optical [56,128], magnetic [55,56,126,127], electrical [99], catalytic [134], self-assembly ability in film and solution [55,99] or some porosity [126,127,135], not found in classical amorphous silicones. In addition, the co-existence in the structure of highly hydrophobic methylsilicone units and somewhat polar coordinated blocks gives the polymer an amphiphile character enabling it, when it is soluble, to reduce the surface tension of the solvent and to self-assemble in solution beyond its specific critical aggregation concentration. These aggregates can be used for modification of a flat surface, making it an intelligent material which can respond to electrical, optical or magnetic stimuli [56].

Depending on the structure and proportion of the silicone component, the coordination polymers obtained have values of water vapor moisture sorption generally below 10 wt.%, namely, between 1.5 [133] and 9.7 wt.% [126]. A higher value, 15.45 wt.% [128] was recorded in the case of the Mn complex with dicarboxylic acid having a diphenylsilane spacer. The lowest values, below 2.6 wt.% [128,134,135], were shown by all complexes with H_2_Cx ligand. The high degree of methylation and the siloxane bond flexibility increase the hydrophobic character of the coordination compounds. The metal-organic frameworks based on 1,3-bis(carboxypropyl)tetramethyldisiloxane with aluminum sulphate showed a moisture sorption capacity of 3.44 wt.%, much lower (about 15 times smaller) compared to the value of 50.06 wt.% recorded for aluminum fumarate prepared and analyzed in similar conditions [129].

## 5. Conclusions and Outlooks

Silicones are amorphous under normal conditions. Their low surface tension makes them easy to form films, which can be further stabilized by crosslinking. This feature, along with others, such as high flexibility and deformability (regularly achieving >300% mechanical breaking strain) and low value of the modulus of elasticity as well as the wide thermal window in which they are stable/functional, and weather and environmental resistance, have made silicones among the select few polymers of choice in the development of DETs. Their performances for such applications can be sought by variations of the structural parameters (molecular mass, polydispersity, crosslinking pattern). Attachment of functional groups or incorporation of active fillers in the silicone matrix followed by crosslinking are other variables that allow adjusting the electromechanical properties of silicone films (especially for increasing dielectric permittivity), but also allow the polymers to respond in different ways (deformation, color, refractive index, etc.) to other external stimuli (temperature, magnetic field, wavelength, etc.). When silicone fragments are co-chained by different interactions with other diorganosiloxane (in which methyl groups are substituted with polar organic or reactive groups), organic, inorganic or mixed segments/blocks, the intermolecular interactions established between the latter can lead to the formation of self-assembled structures, from biphasic (in the case of siloxane and siloxane-organic copolymers) to crystalline (coordination polymers). While silicone films are homogeneous, smooth and respond to stimuli by dimensional change, films based on structures with different degrees of ordering form nanostructures that respond to stimuli (e.g., solvent) by changing morphology. In the case of crystalline structures, siloxane units are not able to participate in the establishment of intermolecular interactions other than hydrophobic ones, due to the extremely non-polar nature of methyl groups attached to silicon. However, the high flexibility of the siloxane motif can allow the adoption of unusual conformations, while its surface activity facilitates orientation of the methyl groups to the air interface, imparting hydrophobic surface behavior to the compound.

The results so far prove the high potential that silicones still have for development based on them, of new compounds and materials designed according to requirements. The perspectives for silicones research in Romania seem promising in our view, since ongoing projects and innovative ideas stimulate the approach of new structures, formulations based on them and original applications. Development of self-healing and/or smart silicone materials with multiple, switchable functions useful for building different transducers and adaptive systems, their optimization for 3D printing, the challenges to obtain highly-ordered siloxane-containing materials, the demonstration of unique applicative potential of siloxane-based surfactants or the sustained concerns for a cleaner environment involving silicone materials, are only a few directions to be developed in the next years.

## Figures and Tables

**Table 1:** Important weather resistances of main classes of dielectric elastomers; where: E—excellent; G—Good; F—Fair; P—Poor [77,78].

Properties	Elastomer Type			
	Silicone	Acrylic	Polyurethane	Natural Rubber
Atmospheric ageing resistance	E	E	F	P
Oxidation resistance	E	E	E	G
Heat resistance	E	E	F–G	F–G
Low temperature flexibility	E	P	F	E
Moisture resistance	E	F	G	G–E

## Data Availability

Data to support statements in this review are available from the corresponding author [Maria Cazacu], upon reasonable request.

## Supplementary Materials

The following are available online at https://www.mdpi.com/article/10.3390/polym13101605/s1, Articles on silicones published by different research groups in Romania (source: web of science; keywords: polydimethylsiloxane; siloxane; silicones); Figure S2: Milestones in the development of silicones in Romania and of “Petru Poni” Institute of Macromolecular Chemistry in Iasi on the background of their evolution worldwide.

## References

[1] Marcu M., Stiubianu G., Vosniuc I., Spiratos M. (1978). Procedure to Obtain Dimethylsiloxane Cycles. Romanian Patent.

[2] Marcu M., Spiratos M., Stiubianu G. (1978). Procedure for the Preparation of the Elastomer Polydimethylsiloxane. Romanian Patent.

[3] Marcu M., Ilie S., Stiubianu G. (1980). Procedure for Obtaining Polydimethylsiloxane-α,ω-Diols. Romanian Patent.

[4] Marcu M., Ilie S., Stiubianu G., Perjoiu M., Roman L.G., Pricop L. (1984). Process for Obtaining One-Component Silicone Rubbers with Room Temperature Vulcanizing. Romanian Patent.

[5] Marcu M., Ilie S., Stiubianu G., Perjoiu M., Roman G., Pricop L., Streba E. (1985). Process for Obtaining Methylvinylsilicone Rubber. Romanian Patent.

[6] Marcu M., Ilie S., Stiubianu G. (1985). Process for obtaining heptamethylvinylcyclotetrasiloxane. Romanian Patent.

[7] Harabagiu V., Cotzur C., Luchian N., Marcu M., Bostan M., Istrate M. (1992). Procedure for Obtaining a Multifunctional Silicone Lubricant. Romanian Patent.

[8] Chelaru N., Bancila M., Marcu M., Luchian N., Angheluta A., Onofrei M. (1995). Process and Installation for Obtaining Continuous Flow of Demoulding Polydimethylsiloxane Oils. Romanian Patent.

[9] Ionescu C., Marcu M., Giurgiu D. (1995). Reactor for the Synthesis of Metilclorsilanilor. Romanian Patent.

[10] Luchian N., Marcu M., Sacarescu L., Rugina T. (1998). Procedure for Obtaining Silicone Greases. Romanian Patent.

[11] Cazacu M., Marcu M. (1995). Silicone Rubbers. Ix. Contributions to polydimethylsiloxane-α,ω-diols synthesis by heterogeneous catalysis. J. Macromol. Sci. A.

[12] Cazacu M., Marcu M., Holerca M.N., Petrovan S., Lazarescu S. (1996). Heterogeneous catalyzed copolymerization of octamethylcyclotetrasiloxane with 1,3,5,7-tetravinyl-1,3,5,7-tetramethylcyclo-tetrasiloxane. J. Macromol. Sci. A.

[13] Cazacu M., Marcu M., Ibanescu C., Petrovan S., Holerca M., Simionescu M. (1996). Cationic heterogeneous copolymerization of octamethylcyclotetrasiloxane with 1,3,5,7-tetramethyl-1,3,5,7-tetravinylcyclotetra-siloxane: Optimization of reaction conditions. Polym. Plast. Technol. Eng..

[14] Cazacu M., Marcu M., Dragan S., Matricala C. (1996). Anionic polymerization of cyclosiloxanes in heterogeneous medium. J. Appl. Polym. Sci..

[15] Cazacu M., Marcu M., Vlad A., Caraiman D., Racles C. (1999). Synthesis of functional telechelic polydimethylsiloxanes by ion-exchangers catalysis. Eur. Polym. J..

[16] Cazacu M., Marcu M., Dragan S., Matricala C., Simionescu M., Holerca M. (1997). Dimethyldiphenylsiloxane copolymers synthesis by ion exchanger catalysis. Polymer.

[17] Cazacu M., Cazacu M. (2008). Polymers containing Si, O and other elements within backbone. Advances in Functional Heterochain Polymers.

[18] Marcu M., Cazacu M., Lazarescu S., Matricala C., Simionescu M., Bolohan S. (2001). Procedure to Obtain Tetramethyl-tetravinylcyclotetrasiloxane. Romanian Patent.

[19] Cazacu M. (2010). Possibilities to develop functional materials on silicone/silica backbones. Recent Developments in Silicone-Based Materials.

[20] Cazacu M., Dragan S. (2005). Polysiloxanes bearing ionic groups. Focus on Ionic Polymers.

[21] Iojoiu C., Abadie M.J.M., Harabagiu V., Pinteala M., Simionescu B.C. (2000). Synthesis and photocrosslinking of benzyl acrylate substituted polydimethylsiloxanes. Eur. Polym. J..

[22] Simionescu C.I., Rusa M., David G., Pinteala M., Harabagiu V., Simionescu B.C. (1997). Block and graft copolymers with polysiloxane and poly(N-acyliminoethylene) sequences. Angew. Makromolek. Chem..

[23] Rosati D., Perrin M., Navard P., Harabagiu V., Pinteala M., Simionescu B.C. (1998). Synthesis of poly(styrene-dimethylsiloxane) block copolymers: Influence of the phase-separated morphologies on the thermal behaviors. Macromolecules.

[24] Cazacu M., Racles C., Dragan S. (2006). Recent developments in siloxane-based polymers and copolymers. New Trends in Nonionic (co)Polymers and Hybrids.

[25] Cazacu M., Vlad A., Racles C., Marcu M. (2003). Incorporation of siloxanes in hydrolytically degradable structures. Eur. Polym. J..

[26] Cazacu M., Vlad A., Simionescu M., Racles C., Marcu M. (2002). Incorporation of the siloxanes in hydrolytically degradable organic structures. II. Segmented siloxaneimide poly(anhydride)s. J. Macromol. Sci. A.

[27] Pislaru-Danescu L., Telipan G., Racles C. (2020). CO2 Concentration Sensor with Sensitive Element Based on Supramolecular Organo-Siloxane Polymer. Romanian Patent.

[28] Cazacu M., Racles C., Vlad A., Antohe M., Forna N. (2009). Silicone-based Composite for Relining of Removable Dental Prosthesis. J. Compos. Mater..

[29] Cazacu M., Ignat M., Racles C., Vlad A., Alexandru M., Zarnescu G. (2009). Polydimethylsiloxane/silica composites incorporating pyrite powders for actuation elements. Polym. Int..

[30] Alexandru M., Cristea M., Cazacu M., Ioanid A., Simionescu B.C. (2009). Composite materials based on polydimethylsiloxane andin situgenerated silica by using the sol-gel technique. Polym. Compos..

[31] Sacarescu L., Kostromin S., Bronnikov S. (2015). Synthesis and properties of polydiphenylsilane/fullerene C_60_ nanocomposites. Mater. Chem. Phys..

[32] Sacarescu L., Mangalagiu I., Simionescu M., Sacarescu G., Ardeleanu R. (2008). Polysilanes. a new route toward high performance EL devices. Macromol. Symp..

[33] Stiubianu G., Racles C., Nistor A., Cazacu M., Simionescu B.C. (2011). Cellulose modification by crosslinking with siloxane diacids. Cellul. Chem. Technol..

[34] Stiubianu G., Cazacu M., Nicolescu A., Hamciuc V., Vlad S. (2009). Silicone-modified cellulose. Crosslinking of the cellulose acetate with 1,1,3,3-tetramethyldisiloxane by Pt-catalyzed dehydrogenative coupling. J. Polym. Res..

[35] Stiubianu G., Racles C., Cazacu M., Simionescu B.C. (2010). Silicone-modified cellulose. Crosslinking of cellulose acetate with poly[dimethyl(methyl-H)siloxane] by Pt-catalyzed dehydrogenative coupling. J. Mater. Sci..

[36] Enescu D., Hamciuc V., Pricop L., Hamaide T., Harabagiu V., Simionescu B.C. (2009). Polydimethylsiloxane-modified chitosan I. Synthesis and structural characterisation of graft and crosslinked copolymers. J. Polym. Res..

[37] Enescu D., Hamciuc V., Ardeleanu R., Cristea M., Ioanid A., Harabagiu V., Simionescu B.C. (2009). Polydimethylsiloxane modified chitosan. Part III: Preparation and characterization of hybrid membranes. Carbohydr. Polym..

[38] Stiubianu G., Cazacu M., Cristea M., Vlad A. (2009). Polysiloxane-lignin composites. J. Appl. Polym. Sci..

[39] Cazacu M., Stiubianu G. (2013). Process for Obtaining a Room Temperature Vulcanization Silicone Rubber. Romanian Patent.

[40] Marcu M., Vlad A. (2010). Procedure to Obtain Diphenylsilanediol in Heterogeneous Catalysis with Anion Exchangers. Romanian Patent.

[41] Tugui C., Tiron V., Dascalu M., Sacarescu L., Cazacu M. (2019). From ultra-high molecular weight polydimethylsiloxane to super-soft elastomer. Eur. Polym. J..

[42] Cazacu M., Vlad A., Alexandru M., Budrugeac P., Racles C., Iacomi F. (2010). Polydimethyldiphenylsiloxanes/silica interconnected networks: Preparation and properties evaluation. Polym. Bull..

[43] Brunchi C.-E., Morariu S., Cazacu M., Bercea M. (2010). Influence of the solvent quality on the thermodynamic behavior of polymethylphenylsiloxane solutions. Ind. Eng. Chem. Res..

[44] Andriot M., Chao S.H., Colas A., Cray S., de Buyl F., DeGroot J.V., Dupont A., Easton T., Garaud J.L., Gerlach E., De Jaeger R., Gleria M. (2007). Silicones in industrial applications. Inorganic Polymers.

[45] Functional Groups 27 April 2019. https://bio.libretexts.org/@go/page/8390.

[46] Noll W. (1968). Chemistry and Technology of Silicones.

[47] Voronkov M.G., Mileshkevich V.P., Yuzhelevskii Y.A., Voronkov M.G., Mileshkevich V.P., Yuzhelevskii Y.A. (1978). The Siloxane Bond.: Physical Properties and Chemical Transformations.

[48] Kuo A.C.M. (1999). Poly(dimethylsiloxane) Polymer Data Handbook.

[49] (2009). Surface Tension and Its Measurement. Adhesives Technology Handbook.

[50] Klykken P., Servinski M., Thomas X. Silicone Film-Forming Technologies for Health Care Applications, Dow Corning White Paper, Literature no. 2-1068A-01 2009. http://citeseerx.ist.psu.edu/viewdoc/download;jsessionid=C84383C5BB2BBA983BF41F08CE6476D6?doi=10.1.1.578.7746&rep=rep1&type=pdf.

[51] Shankar R., Ghosh T.K., Spontak R.J. (2007). Dielectric elastomers as next-generation polymeric actuators. Soft Matter.

[52] Wang N., Cui C., Guo H., Chen B., Zhang X. (2018). Advances in dielectric elastomer actuation technology. Sci. China Technol. Sci..

[53] Tugui C., Stiubianu G.T., Serbulea M.S., Cazacu M. (2020). Silicone dielectric elastomers optimized by crosslinking pattern—A simple approach to high-performance actuators. Polym. Chem..

[54] Zaltariov M.-F., Cazacu M., Racles C., Musteata V., Vlad A., Airinei A. (2015). Metallopolymers based on a polyazomethine ligand containing rigid oxadiazole and flexible tetramethyldisiloxane units. J. Appl. Polym. Sci..

[55] Zaltariov M.-F., Cazacu M., Sacarescu L., Vlad A., Novitchi G., Train C., Shova S., Arion V.B. (2016). Oxime-bridged Mn6 clusters inserted in one-dimensional coordination polymer. Macromolecules.

[56] Shova S., Vlad A., Damoc M., Tiron V., Dascalu M., Novitchi G., Ursu C., Cazacu M. (2020). Nanoscale coordination polymer of dimanganese(II) as infinite, flexible nano-sheets with photo-switchable morphology. Eur. J. Inorg. Chem..

[57] Carpi F., De Rossi D., Kornbluh R., Pelrine R.E., Sommer-Larsen P. (2008). Dielectric Elastomers as Electromechanical Transducers. Fundamentals, Materials, Devices, Models and Applications of an Emerging Electroactive Polymer Technology.

[58] Shankar R., Ghosh T.K., Spontak R.J. (2007). Electroactive Nanostructured Polymers as Tunable Actuators. Adv. Mater..

[59] Koo C.M., El-Sonbati A. (2012). Electroactive thermoplastic dielectric elastomers as a new generation polymer actuators. Thermoplastic Elastomers.

[60] Poplavko Y.M. (2018). Polar dielectrics in electronics. Electronic Materials: Principles and Applied Science.

[61] Ni N., Zhang L., Çankaya N. (2017). Dielectric Elastomer Sensors. Elastomers.

[62] Ignat M., Zarnescu G., Hamciuc E., Hamciuc C., Cazacu M., Sava I. (2016). Microactuator Based on Polymer. Romanian Patent.

[63] Pelrine R., Kornbluh R.D., Eckerle J., Jeuck P., Oh S., Pei Q., Stanford S., Bar-Cohen Y. (2001). Dielectric elastomers: Generator mode fundamentals and applications. Smart Structures and Materials 2001: Electroactive Polymer Actuators and Devices.

[64] Baumgartner R., Keplinger C., Kaltseis R., Schwödiauer R., Bauer S. (2011). Dielectric elastomers: From the beginning of modern science to applications in actuators and energy harvesters. Electroactive Polymer Actuators and Devices (EAPAD) 2011. Proc. SPIE.

[65] Zhao H., Hussain A.M., Israr A., Vogt D.M., Duduta M., Clarke D.R., Wood R.J. (2020). A wearable soft haptic communicator based on dielectric elastomer actuators. Soft Robot..

[66] Moretti G., Rosset S., Vertechy R., Anderson I., Fontana M. (2020). A review of dielectric elastomer generator systems. Adv. Intell. Syst..

[67] Park B.J., Park S., Choi M., Park S.K., Yun S., Shin E., Woong J. (2020). Monolithic focus-tunable lens technology enabled by disk-type dielectric-elastomer actuators. Sci. Rep..

[68] Wang J., Gao D., Lee P.S. (2021). Recent Progress in Artificial Muscles for Interactive Soft Robotics. Adv. Mater..

[69] Bahl S., Nagar H., Singh I., Sehgal S. (2020). Smart materials types, properties and applications: A review. Mater. Today Proc..

[70] Van de Voorde M., de Gruyter W. (2018). Nanoscience and Nanotechnology: Advances and Developments in Nano-sized Materials.

[71] Zhu F., Zhang C., Qian J., Chen W.Q. (2016). Mechanics of dielectric elastomers: Materials, structures, and devices. J. Zhejiang Univ. Sci. A.

[72] Clarson S.J., Semlyen J.A., Englewood Cliffs N.J. (1993). Siloxane Polymers.

[73] Lötters J.C., Olthuis W., Veltink P.H., Bergveld P. (1997). The mechanical properties of the rubber elastic polymer polydimethylsiloxane for sensor applications. J. Micromech. Microeng.

[74] Mata A., Fleischman A.J., Roy S. (2005). Characterization of polydimethylsiloxane (PDMS) properties for biomedical micro/nanosystems. Biomed. Microdevices.

[75] Tugui C., Cazacu M., Sacarescu L., Bele A., Stiubianu G., Ursu C., Racles C. (2015). Full silicone interpenetrating bi-networks with different organic groups attached to the silicon atoms. Polymer.

[76] Bricout N. (1996). Advantages and disadvantages of silicones. Breast Surgery.

[77] Cichosz S., Masek A., Zaborski M. (2018). Polymer-based sensors: A review. Polym. Test..

[78] Xu F., Li X., Shi Y., Li L., Wang W., He L., Liu R. (2018). Recent developments for flexible pressure sensors: A review. Micromachines.

[79] Tugui C., Ursu C., Sacarescu L., Asandulesa M., Stoian G., Ababei G., Cazacu M. (2017). Stretchable energy harvesting devices: Attempts to produce high performance electrodes. ACS Sustain. Chem. Eng..

[80] Bele A., Tugui C., Sacarescu L., Iacob M., Stiubianu G., Dascalu M., Racles C., Cazacu M. (2018). Ceramic nanotubes-based elastomer composites for applications in electromechanical transducers. Mater. Des..

[81] Bele A., Tugui C., Asandulesa M., Ionita D., Vasiliu L., Stiubianu G., Iacob M., Racles C., Cazacu M. (2018). Conductive stretchable composites properly engineered to develop highly compliant electrodes for dielectric elastomer actuators. Smart Mater. Struct..

[82] Bele A., Cazacu M., Neagu M., Popescu M., Racles C., Ioanid G.I. (2020). Modular Installation and Procedure for Obtaining Stratified Polymeric Generators. Romanian Patent.

[83] Zhang J., Sheng J., ONeill C.T., Walsh C.J., Wood R.J., Ryu J.H., Desai J.P., Yip M.C. (2019). Robotic Artificial Muscles: Current Progress and Future Perspectives. IEEE Trans. Robot..

[84] Kussmaul B., Risse S., Wegener M., Bluemke M., Krause J., Wagner J., Feller T., Clauberg K., Hitzbleck J., Gerhard R. (2013). New DEA materials by organic modification of silicone and polyurethane networks. Electroact. Polym. Actuators Devices.

[85] Biedermann M., Blümke M., Wegener M., Krüger H. (2015). Improved actuation strain of PDMS-based DEA materials chemically modified with softening agents. Electroact. Polym. Actuators Devices.

[86] Borayek R., Zhang P., Willy H.J., Zedan M., Zhu J., Ding J. (2020). Programmable, UV-printable dielectric elastomers actuate at low voltage without prestretch and supporting frames. ACS Appl. Electron. Mater..

[87] Opris D.M. (2018). Polar Elastomers as novel materials for electromechanical actuator applications. Adv. Mater..

[88] Racles C., Cazacu M., Fischer B., Opris D.M. (2013). Synthesis and characterization of silicones containing cyanopropyl groups and their use in dielectric elastomer actuators. Smart Mater. Struct..

[89] Racles C., Cozan V., Bele A., Dascalu M. (2016). Polar silicones: Structure-dielectric properties relationship. Des. Monomers Polym..

[90] Racles C., Dascalu M., Bele A., Tiron V., Asandulesa M., Tugui C., Vasiliu A.L., Cazacu M. (2017). All-silicone elastic composites with counter-intuitive piezoelectric response, designed for electromechanical applications. J. Mater. Chem. C.

[91] Racles C., Bele A., Dascalu M., Musteata V.E., Varganici C.D., Ionita D., Vlad S., Cazacu M., Dünki S.J., Opris D.M. (2015). Polar-nonpolar interconnected elastic networks with increased permittivity and high breakdown fields for dielectric elastomer transducers. RSC Adv..

[92] Racles C., Musteata V.E., Bele A., Dascalu M., Tugui C., Matricala A.L. (2015). Highly stretchable composites from PDMS and polyazomethine fine particles. RSC Adv..

[93] Bele A., Cazacu M., Stiubianu G., Vlad S., Ignat M. (2015). Polydimethylsiloxane–barium titanate composites: Preparation and evaluation of the morphology, moisture, thermal, mechanical and dielectric behavior. Compos. B Eng..

[94] Bele A., Cazacu M., Stiubianu G., Vlad S. (2014). Silicone-barium titanate composites with increased electromechanical sensitivity. The effects of the filler morphology. RSC Adv..

[95] Bele A., Stiubianu G., Varganici C.-D., Ignat M., Cazacu M. (2015). Silicone dielectric elastomers based on radical crosslinked high molecular weight polydimethylsiloxane co-filled with silica and barium titanate. J. Mater. Sci..

[96] Bele A., Dascalu M., Tugui C., Iacob M., Racles C., Sacarescu L., Cazacu M. (2016). Dielectric silicone elastomers filled with in situ generated polar silsesquioxanes: Preparation, characterization and evaluation of electromechanical performance. Mater. Des..

[97] Ştiubianu G., Soroceanu A., Varganici C.-D., Tugui C., Cazacu M. (2016). Dielectric elastomers based on silicones filled with transitional metal complexes. Compos. B Eng..

[98] Stiubianu G., Dumitriu A.-M.C., Varganici C.-D., Tugui C., Iacob M., Bele A., Cazacu M. (2016). Changes induced in the properties of dielectric silicone elastomers by the incorporation of transition metal complexes. High Perform. Polym..

[99] Cazacu M., Racles C., Zaltariov M.-F., Dumitriu A.-M.C., Ignat M., Ovezea D., Stiubianu G. (2013). Electroactive composites based on polydimethylsiloxane and some new metal complexes. Smart Mater. Struct..

[100] Iacob M., Sirbu D., Tugui C., Stiubianu G., Sacarescu L., Cozan V., Zeleňáková A., Čižmár E., Feher A., Cazacu M. (2015). Superparamagnetic amorphous iron oxide nanowires self-assembled into ordered layered structures. RSC Adv..

[101] Iacob M., Tugui C., Tiron V., Bele A., Vlad S., Vasiliu T., Cazacu M., Vasiliu A.L., Racles C. (2017). Iron oxide nanoparticles as dielectric and piezoelectric enhancers for silicone elastomers. Smart Mater. Struct..

[102] Dvornic P.R., Jovanovic J.D., Govedarica M.N. (1993). On the critical molecular chain length of polydimethylsiloxane. J. Appl. Polym. Sci..

[103] Ryan K.J., Lupton K.E., Pape P., John V.B. (2000). Ultra‐high‐molecular‐weight functional siloxane additives in polymers. Effects on processing and properties. J. Vynil Addit. Technol..

[104] Racles C., Dascalu M., Bele A., Cazacu M., Gutiérrez T.J. (2020). Reactive and functional silicones for special applications. Reactive and Functional Polymers Volume One.

[105] Guoyong W., Zhiping D., Qiuxiao L., Wei Z. (2010). Carbohydrate-modified siloxane surfactants and their adsorption and aggregation behavior in aqueous solution. J. Phys. Chem. B.

[106] Racles C. (2010). Siloxane-based surfactants containing tromethamol units. Soft Mater.

[107] Zhou X., Zhang D. (2016). Transition from Micelle to Vesicle of a Novel Sugar-Based Surfactant Containing Trisiloxane. Tenside Surfactants Deterg..

[108] Racles C., Hamaide T., Ioanid A. (2006). Siloxane surfactants in polymer nanoparticles formulation. Appl. Organomet. Chem..

[109] Racles C., Silion M., Sacarescu L. (2018). Multi-tasking pyridyl-functionalized siloxanes. Colloids Surf. A Physicochem. Eng. Asp..

[110] Racles C., Mares M., Sacarescu L. (2014). A Polysiloxane Surfactant Dissolves a Poorly Soluble Drug (Nystatin) in Water. Colloids Surf. A Physicochem. Eng. Asp..

[111] Racles C., Cazacu M., Hitruc G., Hamaide T. (2009). On the feasibility of chemical reactions in the presence of siloxane-based surfactants. Colloid Polym. Sci..

[112] Racles C., Iacob M., Butnaru M., Sacarescu L., Cazacu M. (2014). Aqueous dispersion of metal oxide nanoparticles, using siloxane surfactants. Colloids Surf. A Physicochem. Eng. Asp..

[113] Riess G. (2003). Micellization of block copolymers. Prog. Polym. Sci..

[114] Cazacu M., Vlad A., Munteanu G., Airinei A. (2008). Multifunctional materials based on polyazomethines derived from 2,5-dihydroxy-1,4-benzoquinone and siloxane diamines. J. Polym. Sci. A Polym. Chem..

[115] Vlad A., Cazacu M., Munteanu G., Airinei A., Budrugeac P. (2008). Polyazomethines derived from polynuclear dihydroxyquinones and siloxane diamines. Eur. Polym. J..

[116] Zaltariov M.-F., Cazacu M., Shova S., Varganici C.-D., Vacareanu L., Musteata V., Airinei A. (2014). A silicon-containing polyazomethine and derived metal complexes: Synthesis, characterization, and evaluation of the properties. Des. Monomers Polym..

[117] Demus D., Goodby J., Gray G.W., Spiess H.-W., Vill V. (1998). Handbook of Liquid Crystals.

[118] Teyssier D., Boileau S., Jones R.G., Ando W., Chojnowski J. (2000). Liquid crystalline silicon-containing polymers. Silicon-Containing Polymers.

[119] Racles C., Cazacu M. (2008). Siloxane-containing liquid crystalline polymers. Advances in Functional Heterochain Polymers.

[120] Zhang L., Yao W., Gao Y., Zhang C., Yang H. (2018). Polysiloxane-based side chain liquid crystal polymers: From synthesis to structure-phase transition behavior relationships. Polymers.

[121] Racles C., Cazacu M. (2008). Siloxane-containing liquid-crystalline supramolecular polymers: Preparation and study of thermotropic behavior. J. Appl. Polym. Sci..

[122] Racles C., Cozan V. (2014). New siloxane copolymers with pendant azomethine mesogenic units. Rev. Roum. Chim..

[123] Curteanu S., Racles C., Cozan V. (2008). Prediction of the liquid crystalline property for polyazomethines using modular neural networks. J. Optoelec. Adv. Mat..

[124] Racles C. Phase separation and H-bonding trigger LC behavior in siloxane-containing compounds—Unpuplished work (manuscript in preparation).

[125] Zaltariov M.-F., Cazacu M., Ruiz Molina D., van Eldik R. (2020). Coordination compounds with siloxane/silane-containing ligands capable of self-assembly at nano/micro scale in solid state and in solution. Advances in Inorganic Chemistry (Nanoscale Coordination Chemistry).

[126] Cazacu M., Vlad A., Zaltariov M.-F., Shova S., Novitchi G., Train C. (2014). Di- and tetracarboxylic aromatic acids with silane spacers and their copper complexes: Synthesis, structural characterization and properties evaluation. J. Organomet. Chem..

[127] Vlad A., Zaltariov M.-F., Shova S., Novitchi G., Train C., Cazacu M. (2016). Metal–organic frameworks based on tri- and penta-nuclear manganese(II) secondary building units self-assembled by a V-shaped silicon containing dicarboxylate. RSC Adv..

[128] Turcan-Trofin G.-O., Avadanei M., Shova S., Vlad A., Cazacu M., Zaltariov M.-F. (2018). Metallo-supramolecular assemblies of dinuclear Zn(II) and Mn(II) secondary building units (SBUs) and a bent silicon dicarboxylate ligand. Inorg. Chim. Acta.

[129] Cazacu M., Turcan-Trofin G.-O., Vlad A., Bele A., Shova S., Nicolescu A., Bargan A. (2019). Hydrophobic, amorphous metal-organic network readily prepared by complexing the aluminum ion with a siloxane spaced dicarboxylic acid in aqueous medium. J. Appl Polym Sci..

[130] Gavrish S.P., Shova S., Cazacu M., Dascalu M., Lampeka Y.D. (2020). Syntheses and crystal structures of the one-dimensional coordination polymers formed by [Ni(cyclam)]^2+^ cations and 1,3-bis (3-carb oxy prop yl)tetra methyl disiloxane anions in different degrees of deprotonation. Acta Cryst. E.

[131] Gavrish S.P., Shova S., Cazacu M., Lampeka Y.D. (2020). Crystal structure of the one-dimensional coordination polymer formed by the macrocyclic [Ni(cyclam)]^2+^ cation and the dianion of diphenylsilanediylbis(4-benzoic acid). Acta Cryst. E.

[132] Vlad A., Cazacu M., Zaltariov M.-F., Shova S., Turta C., Airinei A. (2013). Metallopolymeric structures containing highly flexible siloxane sequence. Polymer.

[133] Vlad A., Zaltariov M.F., Shova S., Novitchi G., Varganici C.D., Train C., Cazacu M. (2013). Flexible linkers and dinuclear metallic nodes build up an original metalorganic framework. CrystEngComm.

[134] Racles C., Shova S., Cazacu M., Timpu D. (2013). New highly ordered hydrophobic siloxane-based coordination polymers. Polymer.

[135] Vlad A., Cazacu M., Zaltariov M.-F., Bargan A., Shova S., Turta C. (2014). A 2D metal–organic framework based on dizinc coordination units bridged through both flexible and rigid ligands. J. Mol. Struct..

[136] Robin A.Y., Fromm K.M. (2006). Coordination polymer networks with O- and N-donors: What they are, why and how they are made. Coord. Chem. Rev..

