# Supplementary material for: From Amorphous Silicones to Si-Containing Highly Ordered Polymers: Some Romanian Contributions in the Field
Source: Polymers (Basel). 2021 May 16;13(10):1605. doi: 10.3390/polym13101605 (PMC8157024; doi:10.3390/polym13101605)
Supplement: Supplementary file 1 [file polymers-13-01605-s001.zip › polymers-1192356-supplementary.pdf]

polymers

MDPI

Supporting Information

From amorphous silicones to Si-containing highly ordered polymers: some Romanian contributions in the field

Maria Cazacu, Carmen Racles, Mirela-Fernanda Zaltariov, Mihaela Dascalu, Adrian Bele, Codrin Tugui, Alexandra Bargan, George Stiubianu

Department of Inorganic Polymers, "Petru Poni" Institute of Macromolecular Chemistry, Aleea Gr. Ghica Voda 41A, 700487 Iasi, Romania

The history of silicones in Romania began in the early 1970s, a few decades after their discovery. As a result of the state policy at that time, the research has been financed through priority programs and research contracts with industry focused on solving specific problems for key sectors of the economy (energy, construction, electrical engineering, automotive industry, etc.), where silicones fitted very well. Thus, research in this area aimed the development innovative proprietary technologies for obtaining silicone materials. Initially, a few research centers have been designated for the development of research in this direction. The "Petru Poni" Institute of Macromolecular Chemistry, Iași, through its Department of Inorganic Polymers, has systematically approached research on this topic and has developed it over time, being on the top in Romania by number of publications on the field (Figure S1). The research here began with the production of ethyl silicate and an oligomer thereof, Ethyl Silicate 40. The local and global context in which the researches on silicones were carried out in Romania is presented schematically in Figure S2.

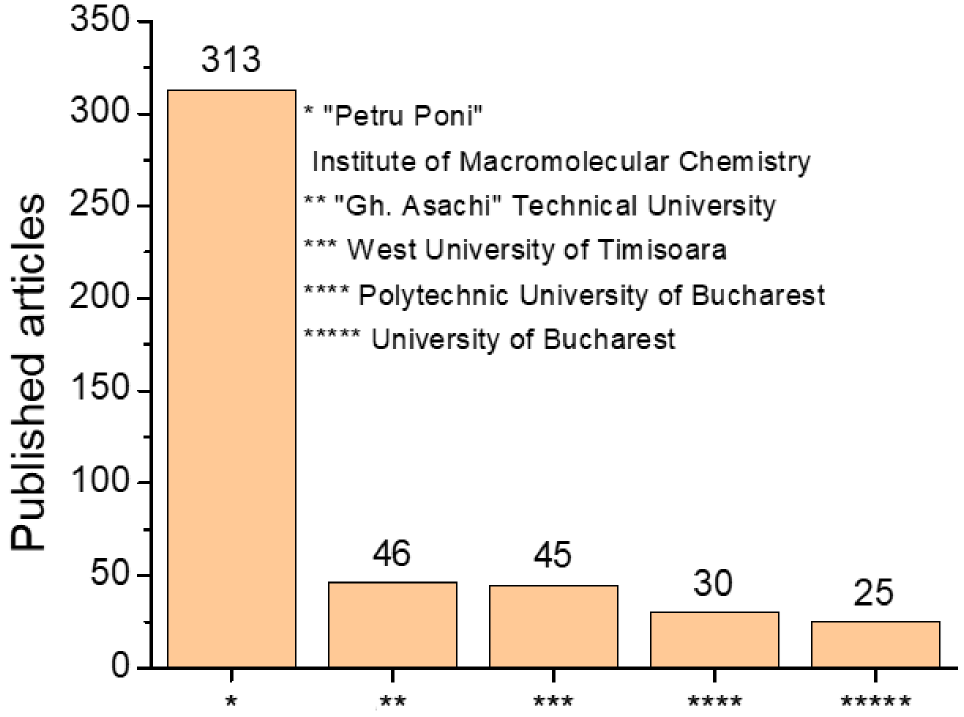

Figure S1. Articles on silicones published by different research groups in Romania (source: web of science; keywords: polydimethylsiloxane; siloxane; silicones).

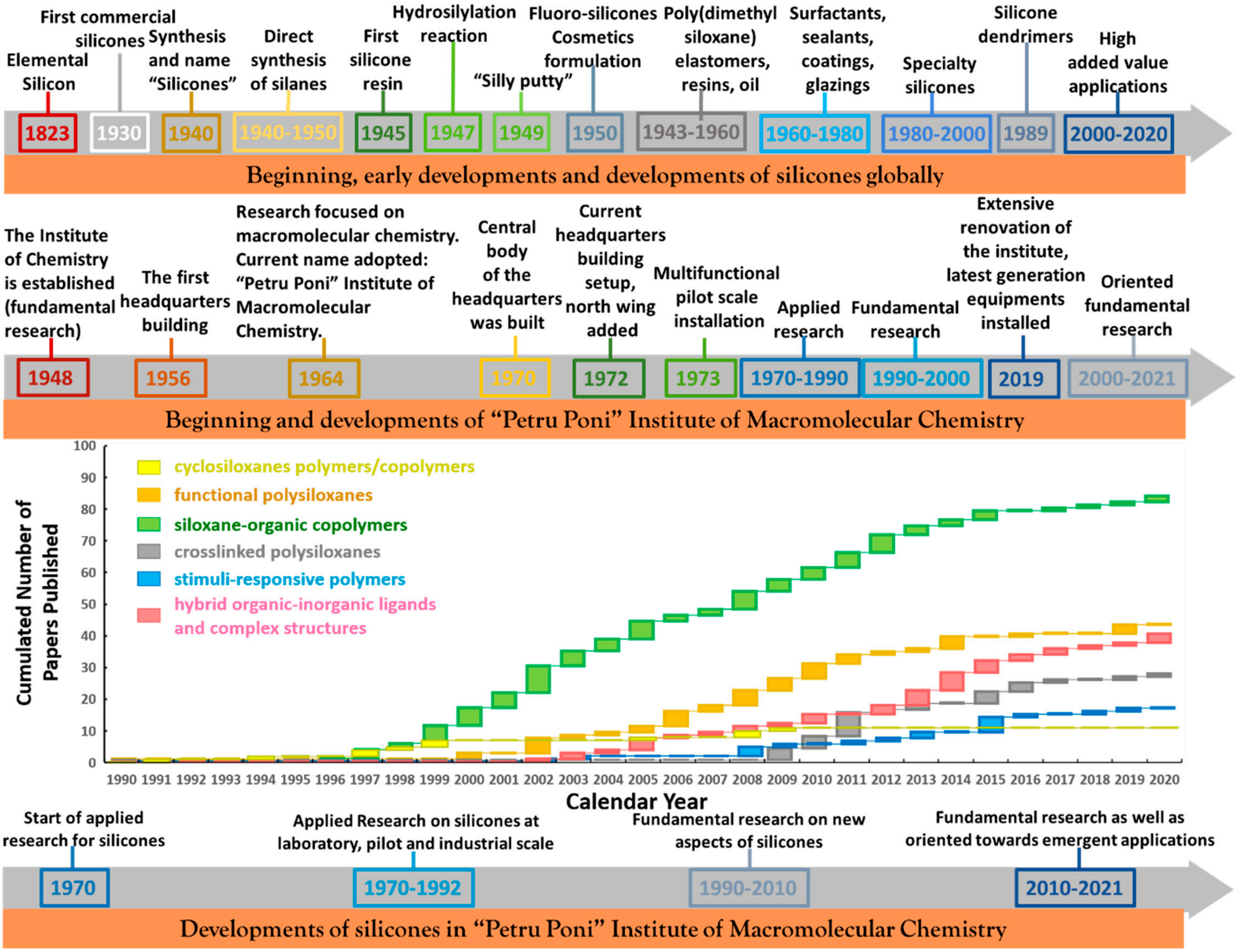

Figure S2. Milestones in the development of silicones in Romania and the development of "Petru Poni" Institute of Macromolecular Chemistry in Iasi on the background of their evolution worldwide.
